# Rare manifestations of cryoglobulinemic vasculitis: a case report

**DOI:** 10.3389/fimmu.2023.1271584

**Published:** 2023-10-13

**Authors:** Charlotte Leleux, Yoann Zerbib, Pierre Pommerolle, Audrey Da Rocha, Marine Serpier, Pauline Caillard

**Affiliations:** ^1^ Department of Internal Medicine, Amiens University Hospital, Amiens, France; ^2^ Department of Intensive Care Unit, University of Picardie Jules Verne, Amiens University Hospital, Amiens, France; ^3^ Department of Nephrology, Dialysis, and Transplantation, University of Picardie Jules Verne, Amiens University Hospital, Amiens, France; ^4^ Department of Hematology, Amiens University Hospital, Amiens, France

**Keywords:** mixed cryoglobulinemic vasculitis, rheumatoid arthritis, cardiac and respiratory involvement, gastrointestinal ischemia, rare and life-threatening organ involvement

## Abstract

**Introduction:**

Cryoglobulinemic vasculitis is a type of small vessel vasculitis diseases that can cause dysfunction in multiple organs. It is characterized by general symptoms, often accompanied by nonspecific cutaneous, articular, neurological, and renal manifestations. Diagnosing cryoglobulinemia through biological testing can be time-consuming and sometimes yields negative results, making diagnosis challenging. There are also other potentially life-threatening complications that can significantly impact prognosis and delay urgent treatment, including digestive manifestations and heart failure.

**Case presentation:**

We report the case of a 60-year-old male patient with a medical history of rheumatoid arthritis. He was admitted to the Nephrology Department for investigation of necrotic vascular purpura, acute kidney injury, and pancytopenia. Laboratory tests revealed consumption of the C3 and C4 complement fractions and the presence of mixed-type III cryoglobulinemia. Despite the initiation of the treatment, the patient rapidly developed multiple severe organ failures, including renal, cardiac, respiratory, and finally digestive complications. Acute colic ischemia led to emergency surgery and the patient was transferred to the Intensive Care Unit. Despite surgical intervention and hemodynamic support, the patient experienced multi-visceral organ failure and died two hours after admission.

**Discussion:**

Mixed cryoglobulinemia vasculitis may result in rare cases of acute and life-threatening organ damage, such as cardiac or respiratory failure with pulmonary hemorrhage, gastrointestinal ischemia, and neurological disorders. These severe manifestations are associated with a poor prognosis and it is crucial to promptly initiate an aggressive therapeutic strategy.

## Introduction

Cryoglobulinemic vasculitis is a rare entity of systemic vasculitis that ubiquitously affects small vessels (1). It can be related to chronic infections, particularly hepatitis C virus (HCV), lymphoproliferative diseases, and connective tissue diseases, such as systemic lupus and rheumatoid arthritis (2, 3). In 25% of cases, the disease is caused by a monoclonal immunoglobulin (type I), but more commonly (75%) it is characterized by a combination of monoclonal or polyclonal immunoglobulins, known as mixed types (II and III) (4, 5). Since the introduction of anti-HCV therapies (HCV accounted for 70-90% of mixed cryoglobulinemia cases (6)) and use of rituximab, the prognosis has significantly improved (7, 8). While a triad of symptoms including purpura, arthralgia, and weakness is present in 30% of symptomatic cryoglobulinemia cases, it is not specific to this type of vasculitis (3, 9). All organs can be affected, some exceptionally, and the combination of severe organ involvement (renal, neurological, cardiac, pulmonary, or digestive) and potentially life-threatening disease is uncommon. Life-threatening cryoglobulinemia is defined as the development of gastrointestinal, pulmonary, cardiac or central nervous system involvement, or kidney failure with biopsy-proven cryoglobulinemic glomerulonephritis (10, 11). Risk factors associated with life-threatening mixed cryoglobulinemia include age over 65, glomerulonephritis, and pulmonary and hepatic disorders (12). We present a case of severe mixed cryoglobulinemia with multiorgan damage in a patient with rheumatoid arthritis.

## Case description

A 60-year-old man was admitted to the Nephrology Department at Amiens University Hospital for acute kidney failure, pancytopenia, and alteration of the general state evolving for several weeks. The patient’s medical history included a diagnosis of seropositive rheumatoid arthritis twenty years ago, with mild symptoms and no previous treatment or follow-up. Additionally, the patient had aortic stenosis and IgG Kappa monoclonal gammopathy under simple medical control and was undergoing withdrawal from alcohol and tobacco addiction.

During the physical examination, necrotic vascular purpura of the lower limbs, a known aortic systolic murmur, signs of portal hypertension with hepatosplenomegaly, and moderate ascites were observed. The patient also displayed conjunctival jaundice, gingival bleeding, and loose teeth. The remaining assessment revealed no arthralgia, other skin lesions, or respiratory or neurological symptoms.

Blood tests revealed acute kidney failure classified as KDIGO 3 (13), with a serum creatinine level of 350 µmol/l, presence of microscopic hematuria, and moderate glomerular proteinuria (550 mg/day). **Table 1** provides further details on the biological characteristics. The autoimmune analysis showed weak positivity for anti-nuclear (AAN) and antineutrophil cytoplasmic (ANCA) antibodies, along with positive anti-B2GP1 and anti-cardiolipin IgM antibodies. Additionally, there was consumption of the C3 and C4 complement fractions and polyclonal hypergammaglobulinemia at 32 g/L. Subsequent testing confirmed the presence of mixed cryoglobulinemia type III, but this result came back positive several days after the initial blood test. Other investigations, including tumor markers, infectious workup (serologies and PCR for human immunodeficiency virus and hepatitis virus B and C, as well as repeated blood cultures), and hematological workup (myelogram and bone marrow biopsy) were negative. Kidney biopsy could not be performed due to severe thrombocytopenia.

**Table 1:** Biological parameters on admission.

Inflammatory parameters Standards		
C-reactive protein (mg/L)	50	[< 5]
Leukocytes (cells/mm^3^)	2800	[4000 – 10000]
Platelets (cells/mm^3^)	45	[150 – 400]
Serum albumin (g/L)	24	[35 – 52]
Kidney involvement		
Serum creatinine (µmol/L)	350	[53-97]
eGFR (mL/min/1.73 m^2^)	16	[> 90]
Serum urea (mmol/L)	17	[3-8]
Urine analysis		
Erythrocytes (cells/mm^3^)	55	[< 10]
Leukocytes (cells/mm^3^)	8	[< 10]
Proteinuria (g/day)	0.6 non-selective	[< 0.2]
Immune parameters		
Cryoglobulin (mg/L)/type	371/Type III polyclonal	
ANCA	1/80	
PR3 (UI/mL)	2.9	
MPO (UI/mL)	0	
Anti-GBM (UI/mL)	0	
Anti-nuclear antibodies	1/160 homogeneous	
Rheumatoid Factor (UI/mL)	21	[< 15]
C3 (g/L)	0.467	[0.9 – 1.7]
C4 (g/L)	0.046	[0.3 – 1.2]
IgG (g/L)	28.7	[7 – 16]
IgM (g/L)	2.3	[0.4 – 2.3]
IgA (g/L)	3.6	[1 – 3]
Serum protein electrophoresis		
Albumine (g/L)	26.3	[40.2-47.6]
Alpha-1 globulin	4.3	[2.1-3.5]
Alpha-2 globulin	7.3	[5.1-8.5]
Beta-1 globulin	2.2	[3.4-5.2]
Beta-2 globulin	3.5	[2.3-4.7]
Gamma globulin	32.5	[8.0-13.5]
Immunofixation electrophoresis	No monoclonal immunoglobulin	

eGFR, estimated glomerular filtration rate; ANCA, antineutrophil cytoplasmic antibodies; MPO, myeloperoxidase; PR3, proteinase 3; GBM, glomerular basement membrane.

## Diagnosis, therapeutic strategy, and patient evolution

**Figure 1:**
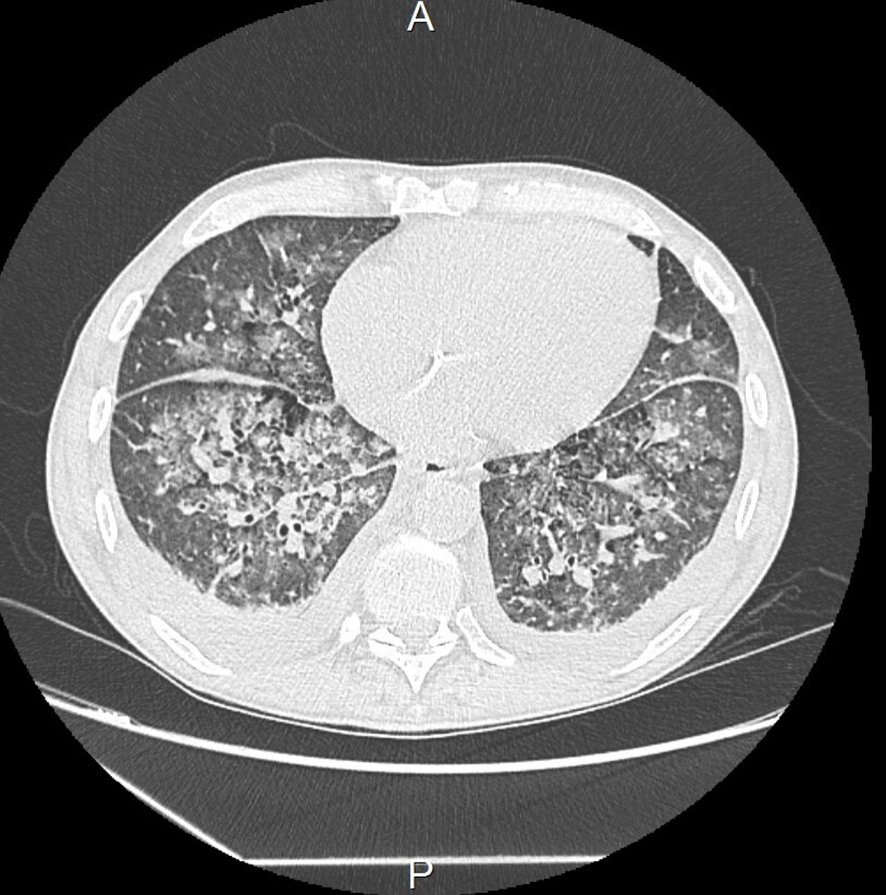
Thoracic computed tomography showing micronodules, condensation lesions, and acute pulmonary oedema.

The patient was initially and rapidly treated with systemic corticosteroid therapy (daily bolus of 500 mg solumedrol for three consecutive days) due to the diagnosis of mixed cryoglobulinemia with severe renal impairment. However, within three days following diagnosis, the patient experienced respiratory distress caused by repeated episodes of flash acute pulmonary oedema (APO), which delayed the initiation of plasma exchange therapy. Hypertension or ischemia was ruled out as the cause through transthoracic echocardiography (TTE). The main hypothesis was capillary hyper-permeability due to small vessel vasculitis. This was supported by the secondary occurrence of ST^+^ acute coronary syndrome with a troponin level of 74.000 ng/L, Sus-ST in leads V4 and V5 of the electrocardiogram, and inferior hypokinesis with preserved ejection fraction on TTE. In this context, coronary angiography revealed nonspecific coronary lesions, confirming the hypothesis of a cryoglobulinemic myocarditis. At the same time, deterioration of kidney function required renal replacement therapy. The exploration of respiratory distress by a thoracic computed tomography showed an APO lesion and small nodular lesions consistent with pneumonia or vasculitis lesions (**Figure 1**). Since there was no clear evidence of an infectious process, such as the absence of fever or increasing inflammatory parameters, antibiotic was not used and plasma exchange therapy and rituximab were started.

**Figure 2:**
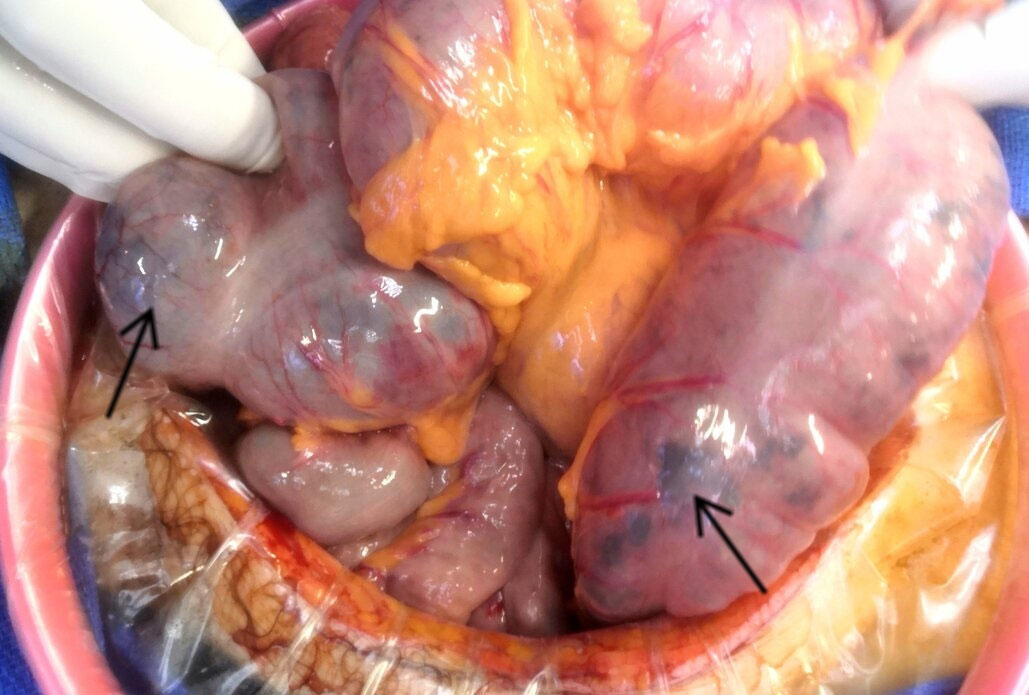
Intraoperative ischemic colon lesions.

Twelve hours later, the patient developed acute colic ischemia with hemodynamic and respiratory failure requiring orotracheal intubation (OTI). Intra-alveolar hemorrhage was suspected due to the presence of blood during OTI. Emergency surgery was performed by ileo-colectomy. Histological analysis of colon samples indicated ischemic lesions without any signs of malignancy, possibly related to vasculitis (**Figure 2**). Unfortunately, the patient died in Intensive Care approximately two hours after surgery due to multi-visceral failure.

## Discussion

Microvascular involvement in cryoglobulinemic vasculitis, particularly when it leads to heart failure, is not well-documented in the literature. Cardiac involvement is rare, affecting 4 to 6% of patients and is not exclusively associated with HCV infection (14). Cardiac involvement generally occurs during renal, skin, and digestive flare-ups (14, 15). These flare-ups can result in severe heart failure due to necrotizing vasculitis lesions of the coronary arteries (16), revealed by thoracic pain or manifestations of congestive heart failure, as described in this case. Most patients do not have previous history of heart disease, suggesting that the underlying vasculitis is in cause. Digestive involvement, occurring in 5% of cases, also involves the arterioles and capillaries and can lead to distal mesenteric vasculitis with a poor prognosis (17). Pulmonary involvement is extremely rare and may be asymptomatic or present as alveolitis with intra-alveolar hemorrhage.

The management of severe forms of cryoglobulinemic vasculitis, which involve life-threatening organ failure, requires aggressive therapy. This typically includes systemic corticosteroid, plasma exchange therapy and the administration of rituximab to eliminate and block immunoglobulin-producing B cells (18). In cases and cohorts described in the literature, this treatment approach has shown effectiveness, although the relatively small patient number studied is a limitation (10, 11).

Furthermore, the underlying pathology should be treated if possible, such as antiviral treatment for HCV, chemotherapy for lymphoproliferative diseases, or immunosuppressive therapy for auto-immune disease (5).

In our case, the patient initially presented with severe kidney failure associated with necrotic purpura and a specific medical history, including rheumatoid arthritis and IgG Kappa monoclonal gammopathy, which raised suspicion of cryoglobulinemic vasculitis. Unfortunately, we were unable to obtain a rapid histological evidence of the vasculitis while waiting for the blood test results. After confirming the diagnosis, specific treatment with rituximab and plasma exchange therapy was delayed due to the immediate need to manage successive organ failure. It is also important to take additional measures, such as limiting exposure to the cold, although this can be challenging in cases of urgent invasive examinations for visceral complications. In this case, the patient experienced severe mixed cryoglobulinemia with an explosive progression of organ failure, affecting successively kidneys, lungs, heart, and digestive system ultimately leading to the patient’s death. This case emphasizes the importance of initiating aggressive treatment promptly when there is a strong suspicion of serious cryoglobulinemic vasculitis, even without biological or histological confirmation of cryoglobulinemia, in order to prevent rapid and potentially fatal evolution. ANCA positivity may also discuss the concomitant diagnosis of ANCA vasculitis, contributing to the clinical symptomatology. The association of these two conditions has already been described, particularly in cases of chronic infection, but the exact mechanisms are not fully understood (19–21). B lymphocytes activation has been suggested to play a role in the interaction between the two types of antibodies. In chronic infection, the presence of plasma cryoglobulin may trigger ANCA-type vasculitis (22). Apart from these rare cases, there may be cross-reactivity between the two types of antibodies, leading to “false positive” ANCA blood test results (19).

The diagnosis of cryoglobulemic vasculitis remains challenging due to its clinical variability, limited knowledge of the underlying mechanisms, and lack of clear diagnostic criteria. Moreover, the cryoglobulin test is subject to rigorous sampling and transport conditions which can affect the accuracy of the results. In our case, the low titer of ANCA, the predominance of complement consumption on C4, and the presence of rheumatoid arthritis strongly suggest the diagnosis of cryoglobulinemic vasculitis, despite the absence of histological evidence. In addition, treatment for either of these two small vessel vasculitis is based, when a life-threatening damage occurs, on high-dose corticosteroid therapy, immunosuppressive therapy and plasma exchange.

## Conclusion

Cryoglobulinemic vasculitis has a poor prognosis due to the etiology of the disease, multiorgan involvement, and severity of organ damage. Microvascular involvement concerns less than 5% of patients and can simultaneously affect organs such as the kidneys, heart, lungs, and digestive tract. The clinical challenge of these acute forms is the rapid onset of multi-organ failure, which necessitates immediate intervention and may delay the specific treatment for vasculitis. However, when there is a suggestive clinical presentation with severe failure of at least one organ, aggressive treatment should be initiated promptly without waiting for biological or histological confirmation.

## Data availability statement

The original contributions presented in the study are included in the article/supplementary material. Further inquiries can be directed to the corresponding author.

## Ethics statement

The studies involving humans were approved by Ethics committee of the University Amiens Hospital (Reference: PI2023_843_0111). The studies were conducted in accordance with the local legislation and institutional requirements. Written informed consent for participation was not required from the participants or the participants’ legal guardians/next of kin in accordance with the national legislation and institutional requirements. Written informed consent was obtained from the patient's next of kin for the publication of this case report.

## Author contributions

PC: Writing – original draft, Writing – review & editing. CL: Writing – original draft, Writing – review & editing. YZ: Writing – review & editing. PP: Writing – review & editing. AD: Visualization, Writing – review & editing. MS: Writing – review & editing.
